# Antecedents and consequences of South African female athletes’ trust in the coach

**DOI:** 10.3389/fspor.2024.1490897

**Published:** 2024-11-06

**Authors:** Bontle Mashilo, Alliance Kubayi

**Affiliations:** Department of Sport, Rehabilitation and Dental Sciences, Faculty of Science, Tshwane University of Technology, Pretoria, South Africa

**Keywords:** performance, competence, justice, athletes, coaching

## Abstract

The purpose of this study was to investigate female South African school athletes’ trust in their coaches in relation to their perceptions of the coaches’ justice, benevolence, integrity, competence, commitment to coach, willingness to cooperate, and performance. A quantitative cross-sectional research design was used in this study. The results showed that there was a large correlation between trust in the coach and the following factors: perceived justice (*r* = .504, *p* < .01), perceived integrity (*r* = .511, *p* < .01), and perceived competence (*r* = .534, *p* < .01). Furthermore, multiple regression analysis results revealed that perceived justice was the only signiﬁcant predictor of trust in the coach as it had a higher beta value (*β* = .17, *p* < .05) than the other variables. This study shows that coaches should demonstrate fairness in their decision-making by providing players with incentives, opportunities to play, friendly relationships, and places of preference.

## Introduction

The coach–athlete relationship (CAR) has been the subject of significant scholarly interest in the realm of sports coaching (1–3). This could be because the relationship between coaches and their athletes is thought to be the most fundamental structure in sport. Without a coach, an athlete might not be able to perform to the best of their abilities, and the coach exists solely to assist the athlete. Coaches and athletes have close bonds based on communication, reciprocity, and mutual trust (4). Previous research on CAR has revealed that trust is a key indicator of a close relationship between coaches and athletes (1, 2). Mayer et al. (5) define trust as “the willingness of a party to be vulnerable to the actions of another party based on the expectation that the other will perform a particular action important to the trustor, irrespective of the ability to monitor or control that other party” (p. 712). Vulnerability here alludes to the risk that could arise if the trustee fails to perform to expectations. For instance, athletes who engage in potentially dangerous sports techniques expose themselves to vulnerability and demonstrate trust through their willingness to take a risk and follow the coach's instructions (6).

When coaches and athletes engage in direct and frequent encounters, athletes will have greater faith in the coach if they feel that the coach will keep their end of the bargain (7). Coaches have significant power over athletes, being able to set goals for them, monitor their training, and manage their playing time (8). Research has found that athletes were more likely to trust a coach who they saw as possessing justice, benevolence, integrity, and competence (6). Justice may be categorised into distributive, procedural, and interactional components (9, 10). In the coaching context, distributive justice refers to the benefits that players look for, such as chances to practise and become experts at their chosen sport, playing time, desired positions and statuses, and so on. Procedural justice can be described as the need for the coach to consistently apply appropriate criteria when allocating rewards to the team members. Interactional justice includes a coach's cordial and courteous interactions with athletes both individually and as a group, outlining the processes involved in reward distribution (6).

Benevolence is the degree to which a coach shows kindness, loyalty to the athlete's interests and welfare, and consideration of the athlete's needs (6, 11). That is, in order for the athlete to have faith in the coach, the athlete needs to think that the coach acts out of kindness towards the athlete rather than out of a desire to benefit personally from the athlete's successes (6). Integrity “entails the ability (of the coach) to both determine, as well as engage in morally correct behaviour regardless of external pressures” (12). There are several examples in sports where coaches have either broken the law, cheated, or pushed their players to do so. When an athlete witnesses that kind of behaviour in their coach, it casts doubt on the coach's integrity and erodes the player's trust. Competence refers to having the necessary skills to perform tasks within a certain field (6). In the relationship between a coach's skill and athletes’ trust in them, coaching competency can be seen as a crucial component. Indeed, studies have found that athletes are more likely to trust a coach who they see as having great coaching skills (7).

Furthermore, empirical evidence has shown that perceived performance is directly impacted by the outcomes (i.e., willingness to cooperate and commitment to coach) of a coach's trust (6). Indeed, previous studies found that increased cooperation and dedication to the leader are also influenced by trust, and these factors ultimately affect performance (6, 13). Therefore, better performance is thus the result of a higher acceptance of the performance norms and a higher level of commitment to the coach (6). Research has demonstrated that trust fosters cooperative behaviour in people, teams, and institutions (5). In sports, athletes’ willingness to cooperate with the coach and other team members in carrying out the coach's instructions is a function of their acceptance of the coach's judgements and guidance. To put it another way, team members who trust their coach are more likely to accept directions and be eager to do as instructed. On the other hand, team members with little faith in the coach are unlikely to collaborate effectively (6).

Research has shown that there are few studies that address the subtleties of how gender oppressions function and whether or not they are being overcome in various sociocultural situations in Africa (14). Although a number of studies have been carried out on athletes’ trust in coaches and its outcomes (4, 6, 7), there is a dearth of data in the context of South Africa, amounting to one study conducted in a South African university setting (13). Their findings showed that athletes’ trust in the coach was mostly explained by perceptions of benevolence, competence, justice, and integrity. Given that many young, aspirational female athletes view sports as a potential professional career trajectory and trust their coach to help them succeed (13), more research in this area is necessary. Furthermore, such a relationship between the coach and athlete that is influenced by many factors including gender should be explored in more depth and more holistically (15). Against this background, the purpose of this study was to investigate female South African school athletes’ trust in their coaches in relation to their perceptions of the coaches’ justice, benevolence, integrity, competence, commitment to coach, willingness to cooperate, and performance.

## Materials and methods

### Research design and sample

A quantitative cross-sectional research design was used in this study. The study's sample consisted of 265 school-going female athletes (M_age_ = 14.55 ± 2.99 years) from four public schools. All of the study's participants were purposefully chosen because they had been involved in sport.

### Research instrument

The Athlete's Trust in Coach Leadership Questionnaire developed by Zhang and Chelladurai (6) was used to collect data. The questionnaire consists of 24 items grouped into the following eight subscales: trust in the leader (2 items), perceived justice (3 items), perceived benevolence (3 items), perceived integrity (4 items), perceived competence (5 items), commitment (2 items), willingness to cooperate (2 items), and perceived performance (3 items). The response format for all of the sub-scales’ items is a 7-point Likert scale ranging from 1 (*strongly disagree*) to 7 (*strongly agr*ee). The overall Cronbach's alpha coefficient value was 0.943, indicating that the questionnaire was reliable (16).

### Data collection procedure

Prior to data collection, the researcher obtained ethics clearance from the Tshwane University of Technology's Faculty Committee for Research Ethics and Research Ethics Committee. The researcher also sought permission to carry out the study from the Department of Basic Education in Pretoria, South Africa. Prior to data collection, parental consent forms were signed, and the participants signed assent forms. The participants were informed that their participation was voluntary, that there would be no repercussions if they stopped participating at any point, and that their responses would be anonymous and confidential. The researcher visited participants in the data-gathering process on prearranged dates at their schools and gave them questionnaires. Participants took eight to ten minutes to complete the questionnaires.

### Data analysis

The data was analysed with the Statistical Package for the Social Sciences (SPSS), version 28. A significance level of 0.05 was chosen. The Pearson correlation coefficient (*r*) was used to examine relationships between variables and a multiple regression analysis was conducted to predict female athletes’ trust in their coach based on their commitment to coach; willingness to cooperate; and perceptions of the latter's justice, benevolence, integrity, competence, and performance. The Cronbach's alpha coefficient was used to examine the instrument's internal consistency.

## Results

Table 1 displays the correlations between trust in the coach and other factors (perceived justice, perceived benevolence, perceived integrity, perceived competence, commitment to coach, willingness to cooperate, and perceived performance). Trust in the coach was significantly correlated with commitment to coach (*r* = .396, *p* < .01), perceived performance (*r* = .405, *p* < .01), willingness to cooperate (*r* = .469, *p* < .01), and perceived benevolence (*r* = .487, *p* < .01), although the correlations were of medium strength. Furthermore, there was a large correlation between trust in the coach and the following factors: perceived justice (*r* = .504, *p* < .01), perceived integrity (*r* = .511, *p* < .01), and perceived competence (*r* = .534, *p* < .01).

**Table 1:** Correlation analysis between studied variables.

	1	2	3	4	5	6	7	8
1. Trust in the coach	–	.504**	.487**	.511**	.534**	.396**	.469**	.405**
2. Perceived justice		–	.600**	.670**	.724**	.387**	.591**	.447**
3. Perceived benevolence			–	.702**	.675**	.426**	.513**	.470**
4. Perceived integrity				–	.688**	.549**	.552**	.586**
5. Perceived competence					–	.579**	.704**	.568**
6. Commitment to coach						–	.548**	.612**
7. Willingness to cooperate							–	.527**
8. Perceived performance								–

** *p* < .01.

Table 2 shows the results of the multiple regression analysis predicting the female athletes’ trust in their coach based on perceived justice, perceived benevolence, perceived integrity, perceived competence, commitment to coach, willingness to cooperate, and perceived performance. The final model was significant [*F*_(7,254)_ = 20.72, *p* < .001] and explained 36% of the total variance in the trust in the coach (adjusted *R^2^* = .35). Perceived justice was the only signiﬁcant predictor of trust in the coach as it had a higher beta value (*β* = .17, *p* < .05) than the other variables.

**Table 2:** Antecedents of trust in the coach.

	B	SE B	*β*	*t*	Sig.
(Constant)	.586	.381		1.537	.126
Perceived justice	.186	.088	.168	2.123	.035*
Perceived benevolence	.150	.081	.140	1.849	.066
Perceived integrity	.123	.100	.105	1.229	.220
Perceived competence	.136	.108	.120	1.269	.206
Commitment to coach	.065	.073	.063	.895	.371
Willingness to cooperate	.104	.077	.100	1.346	.179
Perceived performance	.053	.086	.043	.614	.540

B, unstandardized coefficient; SE B, standard error; β, standardized beta.

* *p* < 0.05.

## Discussion

The findings indicate a strong relationship between trust in the coach and perceived competence. This result corroborates that of Zhang and Chelladurai (6), who discovered that an athlete's faith in their coach is influenced by coaching competency. This study highlights the benefits of coaching competency and athlete trust in the coach-athlete relationship and illuminates how teams view coaching competency. Competency in coaching seems to convey to players that the coach is respectable, reliable, and trustworthy (7). A strong relationship was also found between trust in the coach and perceived integrity. This finding highlights that coaches need to act ethically to earn athletes’ trust.

The results show that trust in the coach was significantly correlated with perceived justice. The results of the multiple regression analysis indicate that among the female athletes, perceived justice was the only factor that predicted their trust in their coaches. This finding supports Zhang and Chelladurai's (6) findings, which suggested that trust in the coach had an impact on perceived justice. When an athlete considers her coach's decision-making to be fair, she is likely to be committed to the team. This suggests that building an equitable and respectful relationship with others is crucial to earning an athlete's trust. By prioritising treating athletes fairly, coaches can cultivate trust and, consequently, enhance athletes’ performance (17).

The findings showed a significant relationship between coaches’ levels of trust and both commitment to the coach and performance improvement. The more committed athletes are to their coach, the more they accept the coach's performance expectations and, consequently, the better their performance is (6). Thus, higher levels of dedication to the coach could increase the likelihood of acceptance and improvements in performance. Trust is a crucial component of high-achieving teams that is rewarded with exceptional performance from athletes. Athletes are more inclined to work hard and try their hardest to fulfil their responsibilities the more committed they are to their coach. Contrastingly, tensions on a team arising from an athlete's insufficient commitment can lower both individual and team performance (17). Lee et al. (4) have reported that the quality of the leader-player relationship being not good can lead to poor results since there is a lack of dedication, trust, and cooperation.

The present data show that trust in the coach was significantly correlated with willingness to cooperate. Athletes with a high level of trust in their coach are more inclined to cooperate with the coach and, as a result, more likely to perform better (17). Furthermore, the findings show that trust in the coach was significantly related to perceived benevolence. This outcome suggests that coaches must demonstrate via suitable leadership actions that they care about the athletes’ well-being as well as the task at hand (6, 13). For athletes to have faith in their coach, they need to think that the coach acts out of kindness towards the athlete rather than purely out of a desire to benefit personally from the athlete's successes.

This study has a few notable limitations. First, its cross-sectional design has inherent limitations because interpersonal trust grows over time and thus conclusions cannot be drawn about the causal relationship between the constructs (6). Second, the athletes were from school settings where competition is low, and the interaction between coaches and athletes was limited by a short season. Third, the study did not include other demographic information variables that might have affected the findings, such as the coach's gender (male or female), the type of sport, individual or team sports, the athletes’ playing experience, and the frequency of training.

## Conclusion

This study highlights significant relationships between trust in the coach and several factors: perceived justice, perceived integrity, and perceived competence. Of all of the perceived characteristics, justice was the only significant factor that predicted female athletes’ trust in their coach. This study demonstrates that coaches should exhibit fairness when making decisions. They can do so by, for example, giving players incentives, chances to play, cordial relationships, and desirable positions and statuses. Coaches could also individualise the care and encouragement they provide to every athlete.

## Data Availability

The raw data supporting the conclusions of this article will be made available by the authors, without undue reservation.

## References

[1] Jowett S Ntoumanis N. The coach–athlete relationship questionnaire (CART-Q): development and initial validation. Scand J Med Sci Sports. (2004) 14:245–57. 10.1111/j.1600-0838.2003.00338.x 15265147

[2] Jowett S. Coaching effectiveness: the coach-athlete relationship at its heart. Curr Opin Psychol. (2017) 16:154–58. 10.1016/j.copsyc.2017.05.006 28813341

[3] Jowett S Arthur C,. Effective coaching: the links between coach leadership and coach-athlete relationship – from theory to research to practice. In: Petrie T MH & Steinfeldt A A J, editors. APA Handbook of Sport and Exercise Psychology. Washington, DC: American Psychological Association (2019). p. 419–49.

[4] Lee S Kwon S Jang D Kwon H. The effect of coach–athlete fit on the coach–athlete relationship in team sport: role of trust in coach. Int J Sports Sci Coach. (2023) 18(4):986–93. 10.1177/17479541231164771

[5] Mayer RC Davis JH Schoolman FD. An integrative model of organizational trust. Acad Manage Rev. (1995) 20:709e35. 10.2307/258792

[6] Zhang Z Chelladurai P. Antecedents and consequences of athlete’s trust in the coach. J Sport Health Sci. (2013) 2:115–21. 10.1016/j.jshs.2012.03.002

[7] Kao SF Hsieh MH Lee PL. Coaching competency and trust in coach in sport teams. Int J Sports Sci Coach. (2017) 12(3):319–27. 10.1177/1747954117710508

[8] Philippe RA Seiler R. Closeness, co-orientation and complementarity in coach–athlete relationships: what male swimmers say about their male coaches. Psychol Sport Exerc. (2006) 7(2):159–71. 10.1016/j.psychsport.2005.08.004

[9] Chelladurai P. Management of Human Resources in Sport and Recreation. 2nd ed. Champaign, IL: Human Kinetics Publishers (2006).

[10] Greenberg J Colquitt JA. Handbook of Organizational Justice. Mahwah, NJ: Lawrence Erlbaum Associates Publishers (2005).

[11] Livnat Y. Benevolence and justice. J Value Inq. (2003) 3:507e15. 10.1023/b:inqu.0000019054.47424.a1

[12] Resick CJ Hanges PJ Dickson MW Mitchelson JK. A cross-cultural examination of the endorsement of ethical leadership. J Bus Ethics. (2006) 63:345e59. 10.1007/s10551-005-3242-1

[13] Zhang Z Surujlal J. The influence of justice, benevolence, integrity, and competence in the coach-athlete relationship in a South African context. Afr J Phys Health Educ Recreat Dance. (2015) 21(1:1):173–85.

[14] Korsakas P Barreira J Tsukamoto MHC Palma B Collet C Galatti LR. Sport pedagogy and feminism: what is known and what is missing? Findings from a scoping review. Phys Educ Sport Pedagogy. (2024):1–17. 10.1080/17408989.2024.2383190

[15] Norman L. Is there a need for coaches to be more gender responsive? A review of the evidence. Int Sport Coach J. (2016) 3(2):192–6. 10.1123/iscj.2016-0032

[16] Tavakol M Dennick R. Making sense of Cronbach’s alpha. Int J Med Educ. (2011) 2:53–5. 10.5116/ijme.4dfb.8dfd 28029643 PMC4205511

[17] Nikbin D Hyun SS Iranmanesh M Foroughi B. Effects of perceived justice for coaches on athletes’ trust, commitment, and perceived performance: a study of futsal and volleyball players. Int J Sports Sci Coach. (2014) 9(4):561–78. 10.1260/1747-9541.9.4.561

